# Mechanism of *Bacillus subtilis* Y61 Promoting the Growth of *Weissella*: Metabolic Interaction Based on Secretion of Arginine and Isovaleric Acid

**DOI:** 10.3390/foods15132266

**Published:** 2026-06-24

**Authors:** Xinyue Wang, Lianqun Wu, Xin Yang, Miao Yang, Yanping Wu, Lixia Pan, Kai Zhong, Hong Gao

**Affiliations:** 1 College of Biomass Science and Engineering, Sichuan University, Chengdu 610065, China; 17729974511@163.com (X.W.); wulianqun23@163.com (L.W.); 18382570120@163.com (X.Y.); 18728208120@163.com (M.Y.); wyp9202@163.com (Y.W.); gao523@hotmail.com (H.G.); 2 National Key Laboratory of Non-Food Biomass Energy Technology, Nanning 530007, China; panlixia@gxas.cn; 3 Guangxi Key Laboratory of Marine Natural Products and Combinatorial Biosynthesis Chemistry, Guangxi Academy of Sciences, Nanning 530007, China

**Keywords:** Sichuan paocai, *Bacillus subtilis*, cross-feeding, transcriptomic analysis, metabolomic analysis

## Abstract

Elucidating the interactions among microbial communities in the Sichuan paocai fermentation system is of great significance for ensuring the safety and quality of paocai. In this study, the interaction between *Bacillus subtilis* Y61 and *Weissella paramesenteroides* (CWP) was preliminarily verified through the culture of CWP using the cell-free supernatant derived from Y61. Building on this, a transwell chamber was employed to spatially isolate the two bacteria. Combined with transcriptomic and metabolomic profiling, the underlying interaction mechanism was revealed. *Weissella paramesenteroides* (CWP) exhibited enhanced growth in the cell-free supernatant of *Bacillus subtilis* Y61, confirming a cross-feeding relationship between the two strains. In the transwell chamber, the promoting effect was most significant when *Weissella paramesenteroides* (CWP) was in the upper compartment and *Bacillus subtilis* Y61 in the lower compartment. Transcriptomic analysis showed that *Weissella paramesenteroides* (CWP) significantly upregulated genes involved in fatty acid synthesis and metabolism while downregulating those related to amino acid anabolism (*p* < 0.05). Metabolomic analysis further revealed that metabolites secreted by *Bacillus subtilis* Y61, including the key metabolites arginine and isovaleric acid, were markedly depleted during co-culture. Exogenous supplementation assays revealed that the combination of 0.1 g arginine and 2 mg isovaleric acid exhibited the strongest growth-promoting effect on *Weissella paramesenteroides* (CWP). Collectively, these results demonstrated that *Bacillus subtilis* Y61 promoted the growth of *Weissella paramesenteroides* (CWP) through cross-feeding *via* the extracellular secretion of the key metabolites arginine and isovaleric acid.

## 1. Introduction

Sichuan paocai is a traditional Chinese fermented vegetable product with a history of consumption spanning thousands of years in China [1]. Owing to its unique flavor, crisp texture, and potential health benefits, paocai is highly favored by consumers and has become an important sector of the Chinese fermented food industry [2]. Traditional paocai fermentation is a typical spontaneous fermentation process driven by indigenous microorganisms originating from raw materials, brine, production utensils, and the surrounding environment [3]. During fermentation, microorganisms undergo dynamic succession and establish complex ecological networks through nutrient competition, metabolite exchange, environmental adaptation, and signaling interactions, thereby shaping the physicochemical properties, flavor profile, and safety of paocai [4]. However, the instability and uncertainty of natural microbial communities often lead to prolonged fermentation periods and inconsistent product quality, which have become major limitations for industrial paocai production [5]. Therefore, understanding the ecological behavior and interaction mechanisms of core microorganisms is of great significance for regulating fermentation processes and improving paocai quality.

Lactic acid bacteria (LAB) are regarded as the dominant functional microorganisms in paocai fermentation because of their strong acidification ability and probiotic potential [6,7]. Previous studies have shown that *Weissella* and *Leuconostoc* are pioneer bacterial genera during the early stage of paocai fermentation, whereas *Lactiplantibacillus* gradually becomes dominant as fermentation progresses and acidity increases [8]. During the early stage of fermentation, *Weissella* and *Leuconostoc* adapt to the microaerophilic environment by synthesizing protective compounds to alleviate oxidative stress [9]. Early colonizing bacteria rapidly metabolize carbohydrates through heterolactic fermentation pathways, producing organic acids, carbon dioxide, ethanol, and other metabolites that reshape the fermentation environment and drive microbial succession [10]. In particular, *Weissella* species play crucial roles in acid production, flavor precursor formation, exopolysaccharide synthesis, and nitrite degradation during early fermentation. Inoculation with *Weissella* strains has been reported to accelerate acidification and reduce nitrite accumulation in paocai fermentation systems [11]. Nevertheless, the ecological functions of *Weissella* are not solely determined by its individual metabolic capacity, but are also closely associated with interactions among coexisting microorganisms [12].

Recent studies increasingly suggest that microbial interactions are fundamental to the stability and functionality of fermented food ecosystems [13]. In spontaneous fermentation systems, microorganisms rarely function independently; instead, they establish cooperative or competitive relationships through metabolic cross-feeding, nutrient exchange, quorum sensing, and environmental modification [14]. These interactions directly influence microbial community assembly, fermentation efficiency, and metabolite formation. For example, typical LAB can be isolated from traditional fermented foods, while non-LAB strains with promising antimicrobial activity can also be identified [15]. Compared with dominant LAB, non-dominant microorganisms in paocai fermentation have received relatively limited attention, despite their potential importance in regulating community dynamics and fermentation outcomes. Among these microorganisms, *Bacillus* species are frequently detected during paocai fermentation and are considered important contributors to enzymatic hydrolysis, flavor compound formation, and ecological regulation [16,17]. *Bacillus* can produce various extracellular enzymes [18], amino acids, peptides, and volatile metabolites [19], which may alter nutrient availability and environmental conditions, thereby affecting the growth and metabolism of neighboring microorganisms. However, the interaction mechanisms between *Bacillus* and *Weissella* in paocai fermentation ecosystems remain largely unclear.

The application of starter cultures has become an important strategy for improving the controllability and standardization of paocai fermentation [20]. However, the effectiveness of starter cultures depends not only on their individual fermentation characteristics but also on their ecological compatibility and interaction capacity within microbial communities. Therefore, elucidating the interaction mechanisms between functional microorganisms is essential for the rational design of synthetic microbial consortia and the development of efficient fermentation starters. In recent years, integrated multi-omics approaches, particularly transcriptomics and metabolomics, have emerged as powerful tools for investigating microbial interactions in fermented foods [21,22,23]. These technologies enable systematic characterization of gene expression regulation and metabolite exchange during microbial co-culture, thereby providing mechanistic insights into interspecies cooperation and adaptation. Nevertheless, studies employing integrated omics approaches to investigate microbial interaction mechanisms in paocai fermentation are still scarce.

In our previous study, inoculation of *Bacillus subtilis* Y61 significantly improved paocai quality and safety while shortening the fermentation period. Notably, microbial community analysis demonstrated that inoculation with *Bacillus subtilis* Y61 markedly promoted the enrichment of *Weissella* during the early fermentation stage [24]. This observation suggested the existence of a potential cooperative interaction between *Bacillus* (non-LAB) and *Weissella* (LAB). To further elucidate the underlying mechanism, *Weissella paramesenteroides* CWP isolated from paocai was co-cultured with *Bacillus subtilis* Y61 in the present study. A transwell co-culture system was employed to investigate their interaction under non-contact conditions, while transcriptomic and metabolomic analyses were conducted to characterize changes in gene expression and metabolic profiles during co-culture. This study aims to reveal the ecological interaction mechanism between *Bacillus* and *Weissella* and to provide a theoretical basis for the targeted regulation of microbial communities and the rational construction of synthetic starter cultures for paocai fermentation.

## 2. Materials and Methods

### 2.1. Chemicals and Reagents

Arginine and isovaleric acid were purchased from Aladdin Bio-Technology Co., Ltd. (Shanghai, China). Other materials, including MRS agar medium and MRS broth, were obtained from Qingdao Hope Bio-Technology Co., Ltd. (Qingdao, China), while nutrient agar (NA) was purchased from Beijing Aoboxing Bio-technology Co., Ltd. (Beijing, China).

### 2.2. Strains and Strain Identification

*Weissella* strain CWP was obtained from mid-fermentation paocai (CICC 24447). *Weissella* CWP was activated on MRS agar medium. DNA was extracted using the TSINGKE Plant DNA Extraction Kit (Universal, Beijing, China). The extracted DNA sample was appropriately diluted and used as a PCR template. The 16S rDNA sequence was amplified using primers 27F (GAGAGTTTGATCCTGGCTCAG) and 1492R (TACGGCTACCTTGTTACGAC) (TSINGKE, Beijing, China). The amplified product was sequenced, and the sequencing results were assembled using ContigExpress 9.1, with the inaccurate regions at both ends removed. The assembled sequence was subjected to BLAST (https://blast.ncbi.nlm.nih.gov) alignment in the NCBI database, and phylogenetic analysis was performed using the neighbor-joining method in MEGA 12.0 software.

### 2.3. Preparation of Y61 Cell-Free Supernatant

Y61 was preserved in glycerol at −20 °C in a laboratory in Chengdu, China (NCBI GenBank accession No: MN744690). Y61 was activated on nutrient agar (NA) and incubated at 37 °C for 24 h. To eliminate the interference of the culture medium with the experimental results, Y61 was inoculated into MRS broth and incubated at 37 °C for 36 h with shaking (120 rpm). The bacterial concentration was adjusted to approximately 10^8^ CFU/mL. An inoculum of 1% (*v*/*v*)was transferred into fresh MRS broth and incubated at 37 °C for 10 h and 120 rpm. The culture was centrifuged at 6000× *g* for 15 min at 4 °C, and the resulting supernatant was filtered through a 0.22 μm membrane to obtain the cell-free supernatant (CFS) [25].

### 2.4. Cultivation and Inoculation of CWP

*Weissella* strain CWP, stored in glycerol at −20 °C, was cultured on MRS agar at 37 °C for 24 h, then inoculated into MRS broth and incubated at 37 °C for 14 h with shaking at 120 rpm. Then, 1% (*v*/*v*) of the inoculum, adjusted to approximately 10^8^ CFU/mL, was transferred into CFS and fresh MRS broth, respectively. The OD_600_ and pH were measured at 1 h intervals, with three independent replicates per time point.

### 2.5. Transwell Cocultures

To further investigate the promoting effect of Y61 on CWP, a transwell culture system (6-well plate; Costar, Kennebunk, ME, USA) was used. Each well contained an insert with a 0.4 μm membrane at the bottom, allowing material exchange between the upper and lower compartments without direct physical contact. Y61 and CWP were cultured in the upper (1.5 mL MRS) and lower (2.6 mL MRS) compartments, respectively. A co-culture experiment with direct physical contact was also conducted, with a total culture volume of 4.1 mL [26]. To better simulate the interaction between the two strains in the paocai fermentation environment, the inoculation ratio of Y61 to CWP was fixed at 1:5 and 1:10. The addition concentration of Y61 was kept constant. OD_600_ of each compartment and plate counting were measured in triplicate every 12 h. Meanwhile, three pure culture groups of Y61 and CWP were established under two inoculation ratios, with the same inoculation concentrations as those used in the transwell inserts, in a total volume of 4.1 mL. Three randomly assigned wells per group were used as replicates.

### 2.6. Transcriptome Sequencing

CWP cells from monoculture and from the upper chamber of physical contact-free co-culture under both inoculation ratios were collected after 24 h for transcriptomic analysis. RNA-seq library preparation and sequencing were performed by Applied Protein Technology following the manufacturer’s standard protocol.

#### 2.6.1. Total RNA Extraction

Total RNA of CWP cells was extracted using TRIzol reagent (Ambion, Austin, TX, USA). The concentration and purity of the total RNA were determined using a NanoDrop 2000 spectrophotometer (Thermo Scientific, Waltham, MA, USA), and RNA integrity was assessed by agarose gel electrophoresis specifically for RNA. Only RNA samples with a RIN greater than 7 were used for subsequent library construction. The RIN of CWP is shown in Table S1.

#### 2.6.2. Library Development and Quality Assurance

The Zymo-Seq RiboFree Total RNA Library Kit was used to remove ribosomal RNA (rRNA) from the total RNA, and then controlled heating conditions enabled fragmentation by divalent cations. cDNA was synthesized from RNA using random oligonucleotide primers. After purification of the double-stranded cDNA, end repair was performed, an “A” base was added to the 3′ ends, and the products were ligated to sequencing adapters. cDNA fragments of approximately 400–500 bp were selected using AMPure XP beads(Beckman Coulter, Brea, CA, USA). After PCR amplification of the selected fragments, the resulting products were re-purified using AMPure XP beads to generate the final library. Library quality was assessed using the Agilent 2100 Bioanalyzer with the Agilent High Sensitivity DNA Kit (Agilent, 5067-4626, Santa Clara, CA, USA). The total concentration of the library was determined using the PicoGreen assay (Quantifluor-ST fluorometer, Promega, E6090, Madison, WI, USA; Quant-iT PicoGreen dsDNA Assay Kit, Invitrogen, P7589, Waltham, MA, USA), while the effective library concentration was quantified by qPCR using a Thermo Scientific StepOnePlus Real-Time PCR System.

#### 2.6.3. Data Quality Control

Following sequencing on the platform, the resulting image files were processed with the platform’s proprietary software to generate raw sequencing data in FASTQ format (raw data). The raw sequencing data contained adapter sequences and low-quality reads, which could significantly interfere with subsequent bioinformatics analysis. Therefore, further filtering of the sequencing data was required. The quality control criteria were as follows: (1) adapter sequences at the 3′ ends were trimmed using fastp (version 0.22.0); (2) reads with an average quality score below Q20 were discarded. All downstream analyses were performed using high-quality clean data [27].

### 2.7. Transcriptome Data Analysis

Read counts mapped to each gene were quantified using HTSeq (version 0.9.1) to obtain the raw expression level of each gene. To enable comparison of gene expression levels across different genes and samples, the expression values were normalized using FPKM (Fragments Per Kilobase of transcript per Million mapped fragments) [28]. FPKM represents the number of fragments per kilobase of transcript length per million mapped fragments. For paired-end sequencing, each fragment corresponds to two reads, and FPKM only counts fragments where both reads map to the same transcript. DESeq software (version 1.38.3) was employed to carry out differential expression analysis between pairwise comparisons. Genes with an absolute log_2_ Fold Change (|log_2_FoldChange|) > 1 and an FDR < 0.05 were considered significantly differentially expressed [29].

### 2.8. Untargeted Metabolomics

We selected the culture supernatants from pure cultures and physical contact-free co-cultures after 24 h of incubation under two different ratios for metabolomics analysis. Samples were slowly thawed at 4 °C, and an appropriate amount of each sample was added to a pre-chilled methanol/acetonitrile/water solution (2:2:1, *v*/*v*/*v*), followed by vortex mixing. After low-temperature ultrasonication for 30 min, the mixture was left to stand at −20 °C for 10 min, followed by centrifugation at 14,000× *g* and 4 °C for 20 min. The supernatant was collected and dried under vacuum. Before mass spectrometry analysis, the residue was reconstituted in 100 μL of acetonitrile/water (1:1, *v*/*v*), then vortexed and centrifuged at 14,000× *g* and 4 °C for 15 min. The resulting supernatant was taken for injection and analysis. Separation was carried out on a HILIC column installed in a Vanquish LC ultra-high performance liquid chromatography (UHPLC) system. The column temperature was maintained at 25 °C, the flow rate was set at 0.5 mL/min, and the injection volume was 2 μL. The mobile phases consisted of A (water with 25 mM ammonium acetate and 25 mM ammonium hydroxide) and B (acetonitrile). Samples were analyzed in a randomized order without interruption to prevent potential fluctuations in instrument detection signals. System stability and experimental data reliability were monitored and evaluated by interleaving quality control (QC) samples throughout the sample queue. Following separation using a Vanquish LC ultra-high performance liquid chromatography (UHPLC) system, mass spectrometric analysis was performed on an Orbitrap Exploris™ 480 mass spectrometer (Thermo Fisher Scientific) using electrospray ionization (ESI) in both positive and negative ion modes. The ESI source and mass spectrometry parameters were set as follows: nebulizer gas and auxiliary heating gas 1 (Gas 1):50; auxiliary heating gas 2 (Gas 2):2; ion source temperature: 350 °C; spray voltage (ISVF): 3500 V in positive ion mode and 2800 V in negative ion mode. The MS1 mass-to-charge ratio detection range was 70–1200 Da, with a resolution of 60,000 and a scan accumulation time of 100 ms. For MS/MS analysis, a segmented data-dependent acquisition method was used, featuring a scan range of 70–1200 Da, a resolution of 60,000, a scan accumulation time of 100 ms, and a dynamic exclusion time of 4 s.

### 2.9. Metabolomics Data Analysis

Raw data were converted to the mzXML format using ProteoWizard (3.0.19254), followed by peak alignment, retention time correction, and peak area extraction using XCMS software (4.10.0). Metabolite structural identification and data preprocessing were performed on the XCMS-extracted data. This included filtering out missing values (removing ion peaks with >50% missing values), imputing remaining missing values using K-nearest neighbors (KNN), and removing features with a relative standard deviation (RSD) greater than 50%. Subsequently, the quality of the experimental data was evaluated, followed by data analysis.

Metabolites in biological samples were structurally identified by matching their retention times, molecular masses (with a mass error < 10 ppm), tandem mass spectrometry (MS/MS) fragmentation patterns, and collision energies against an in-house database. The identification results were then subjected to rigorous manual re-checking and confirmation. All identified metabolites achieved a confidence level of at least Level 2. All identified metabolites (combining those identified in positive and negative ion modes) were classified and statistically analyzed according to their Chemical Taxonomy classification information. Using the fuzzy c-means (FCM) algorithm from the Mfuzz package, metabolites were clustered into distinct expression modules according to their expression trends. Principal component analysis (PCA), partial least squares discriminant analysis (PLS-DA), and orthogonal partial least squares discriminant analysis (OPLS-DA) were performed on the metabolites. To avoid overfitting of the supervised model during the modeling process, a permutation test was performed to validate the model and ensure its validity. Significantly differential metabolites were screened based on the criteria of OPLS-DA variable importance in projection (VIP) > 1.0, Student’s *t*-test *p* < 0.05, and fold change > 1.0 or <1.0.

### 2.10. Exogenous Supplementation of Arginine and Isovalerate

The addition concentration gradients of arginine and isovaleric acid were determined according to the maximum arginine production yield of the microorganism and the isovaleric acid supplementation level, as reported in the literature [30,31]. Heat-stable substances were added to the MRS broth before autoclaving, while heat-labile substances were introduced only after the sterilized broth had cooled and were then filtered through a 0.22 μm membrane filter. The culture was inoculated with 1% (*v*/*v*) of a 15 h pre-culture of CWP and incubated at 37 °C with shaking at 120 rpm. The OD_600_ of the culture was measured every 1 h.

## 3. Results

### 3.1. Identification and Analysis of Weissella Strain CWP

**Figure 1:**
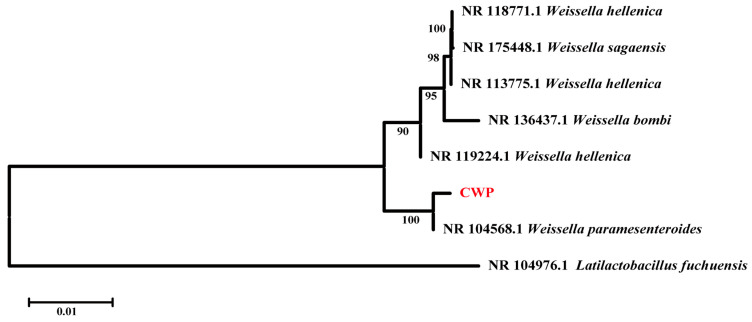
Neighbor-joining phylogenetic tree of *Weissella* strain CWP.

The phylogenetic analysis result of CWP is shown in Figure 1. CWP exhibited the closest genetic relatedness to *Weissella paramesenteroides* and was therefore identified as *Weissella paramesenteroides*.

### 3.2. Y61-Mediated Cross-Feeding of CWP

**Figure 2:**
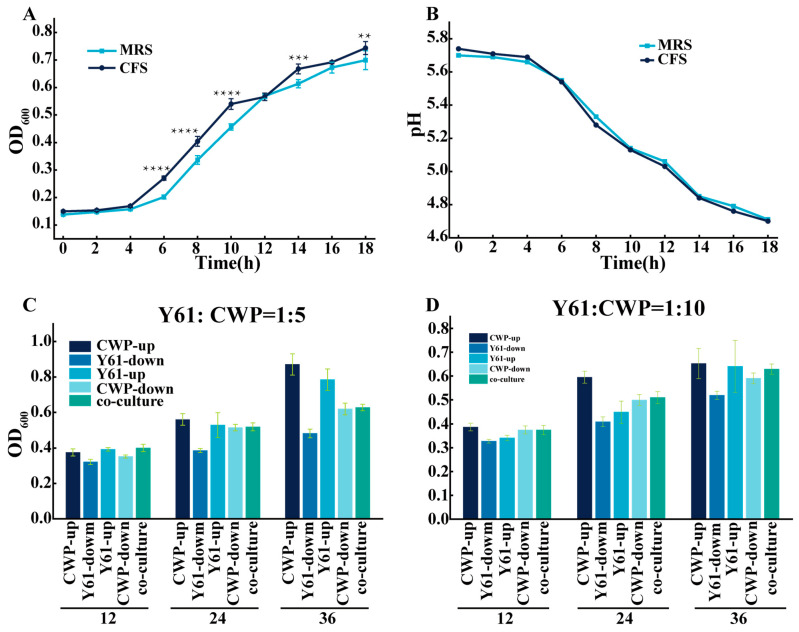
Results of the co-culture of Y61 and CWP. (**A**) OD_600_ changes in CWP cultured in CFS (** *p *< 0.01, *** *p *< 0.001, **** *p *< 0.0001). (**B**) pH changes in CWP cultured in CFS. (**C**,**D**) OD_600_ of mono- and co-culture of Y61 and CWP at 12 h, 24 h, 36 h.

The growth of strain CWP in the CFS of Y61 (as indicated by OD_600_) and the changes in culture pH are shown in Figure 2A,B. After 4 h of cultivation, CWP reached the logarithmic phase. The OD_600_ of CWP in the CFS was consistently higher, and the pH slightly lower, than those in MRS. These results suggested that CWP could grow in the cell-free supernatant of Y61, likely by utilizing the metabolites produced by Y61, thereby leading to accelerated growth. Moreover, the transwell experiment dynamically demonstrated the feeding process of Y61 to CWP (Figure 2C,D). When the inoculation ratio was Y61:CWP = 1:5 and CWP was placed in the upper chamber with Y61 in the lower chamber, the OD_600_ of CWP at 36 h was significantly higher than that in the co-culture control, whereas the OD_600_ of Y61 showed no significant change. Conversely, when CWP was placed in the lower chamber and Y61 in the upper chamber, the OD_600_ of CWP at 36 h remained comparable to that in the co-culture control, while the OD_600_ of Y61 was significantly higher than that in the co-culture control. At an inoculation ratio of Y61:CWP = 1:10, the following growth patterns were observed under different transwell configurations. When CWP was placed in the upper chamber and Y61 in the lower chamber, the OD_600_ of CWP at 36 h was slightly higher than that in the co-culture control and also slightly higher than its value at 24 h. Consistent with this trend, the viable cell count at 36 h was also higher than that at 24 h (Table S2). In contrast, the OD_600_ of Y61 under this configuration was significantly lower than that in the co-culture control, but remained significantly higher than its value at 24 h. When the positions were reversed (CWP in the lower chamber, Y61 in the upper chamber), the OD_600_ of CWP at 36 h was lower than that in the co-culture control, yet higher than its value at 24 h. Meanwhile, the OD_600_ of Y61 showed no significant difference from the co-culture control, but was markedly higher than its value at 24 h. This might be explained by the relatively oxygen-rich condition in the upper chamber. At 48 h, abnormally high OD_600_ values were observed for both CWP and Y61 in the upper chamber. This was likely due to the evaporation of the culture medium (initial volume 1.5 mL) during the 37 °C incubation, which led to an increase in cell concentration per unit volume and consequently resulted in overestimated OD_600_ readings. Therefore, the 48 h data were excluded from the final analysis. At 36 h, both CWP and Y61 remained actively growing. CWP consistently showed higher OD_600_ in the upper chamber than in the lower chamber, where its OD_600_ was comparable to the co-culture control, irrespective of the inoculation ratio. Plate counting (Table 1) confirmed that the elevated OD_600_ of CWP in the upper chamber reflected higher cell density rather than an artifact caused by the smaller volume of the upper chamber. Together, these results demonstrated that Y61 growing in the lower chamber might release metabolites that diffused across the 0.45 μm membrane into the upper chamber, where CWP may directly utilize them to enhance its growth.

### 3.3. Statistics of Differential Genes and Pathway Enrichment Analysis of CWP

**Figure 3:**
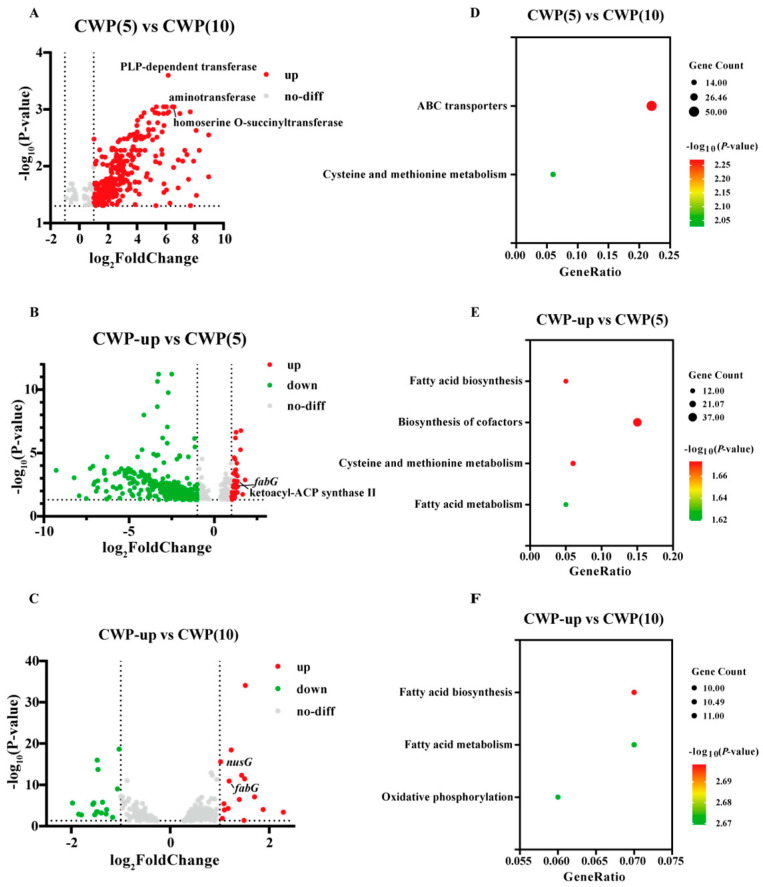
Transcriptomic analysis results of CWP. (**A**) Volcano plot of differentially expressed genes for the two CWP monoculture groups. (**B**) Volcano plot of differentially expressed genes between CWP under physical contact-free co-culture at a 1:5 ratio and CWP under monoculture. (**C**) Volcano plot of differentially expressed genes between CWP under physical contact-free co-culture at a 1:10 ratio and CWP under monoculture. (**D**–**F**) Bubble chart of significantly enriched pathways identified by KEGG analysis of differentially expressed genes.

To elucidate the molecular mechanism underlying CWP’s utilization of Y61 metabolites, we collected CWP cells grown in mono-culture for 24 h and performed transcriptomic analysis. By normalizing the raw read counts, calculating the probability (*p*-value) using a statistical model, and finally performing multiple hypothesis testing correction, genes with significantly differential expression levels were identified. The volcano plots in Figure 3A–C visually displayed the distribution of differentially expressed genes for each comparison pair. These results demonstrated that the gene expression of CWP was significantly influenced by both the inoculation ratio and the culture conditions. To perform functional analysis of these differentially expressed genes, KEGG pathway enrichment analysis was conducted using a significance threshold of FDR < 0.05 (Figure 3D–F). As shown in Figure 3A, CWP significantly upregulated genes involved in amino acid metabolism, including PLP-dependent transferase, homoserine O-succinyltransferase and aminotransferase, and these differentially expressed genes were significantly enriched in the ABC transporters and cysteine and methionine metabolism pathways (Figure 3D), suggesting that CWP upregulated genes involved in amino acid utilization, possibly due to the abundant amino acids present in the external environment. As shown in Figure 3B,C, CWP-up significantly upregulated fatty acid metabolism-related genes, including *fabG* and the ketoacyl-ACP synthase III-encoding gene, which were also significantly enriched in the fatty acid biosynthesis and fatty acid metabolism pathways (Figure 3E,F). KEGG fatty acid biosynthesis pathway analysis further elucidated the functional roles of these genes within this pathway. *FabG* encodes 3-oxoacyl-[acyl-carrier protein] reductase, which serves as a key rate-limiting enzyme in long-chain fatty acid synthesis and plays a central regulatory role in the elongation cycle. Ketoacyl-ACP synthase III catalyzes the initial condensation reaction, thereby initiating fatty acid biosynthesis. Notably, the significantly differentially expressed genes of CWP-up at a Y61:CWP ratio of 1:5 were additionally enriched in amino acid metabolism-related pathways (Figure 3E). These findings indicated that CWP exhibited robust amino acid metabolic activity during the early growth phase, whereas fatty acid metabolism gradually increased once a certain level of biomass was achieved, when CWP was co-cultured with Y61.

### 3.4. Metabolomics Analysis

To identify the key metabolites involved in cross-feeding from Y61 to CWP, we performed metabolomics analysis on the culture supernatants of Y61 and CWP in monoculture, as well as on the culture supernatant collected from the transwell chamber. Figures S1 and S2 show the permutation test plots of the OPLS-DA models for the experimental comparison groups. As the permutation retention rate gradually decreased, both R^2^ and Q^2^ of the permuted models declined progressively, indicating that the original models were not overfitted and exhibited good robustness (*n* = 200). Using strict criteria of OPLS-DA VIP > 1.0, FDR < 0.05, and fold change > 1.0 or <−1.0, we identified significantly differential metabolites with confirmed identities. These metabolites were then subjected to hierarchical clustering analysis and KEGG pathway enrichment analysis.

#### 3.4.1. Metabolomics Analysis of CWP

**Figure 4:**
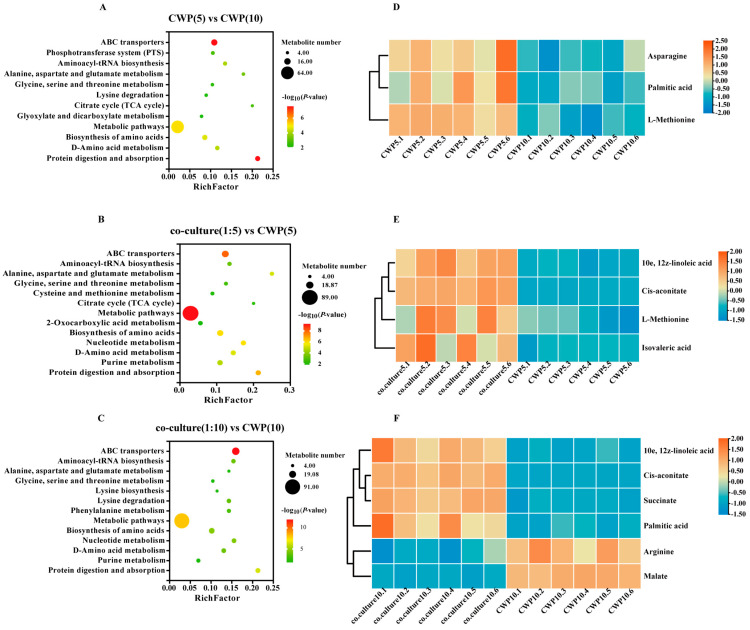
Metabolomics analysis results of CWP. (**A**) Bubble chart of significantly enriched pathways identified by KEGG analysis of differential metabolites between the two CWP monoculture groups. (**B**) Bubble chart of significantly enriched pathways identified by KEGG analysis of differential metabolites between CWP under physical contact-free co-culture at a 1:5 ratio and CWP under monoculture. (**C**) Bubble chart of significantly enriched pathways identified by KEGG analysis of differential metabolites between CWP under physical contact-free co-culture at a 1:10 ratio and CWP under monoculture. (**D**) Hierarchical clustering heatmap of significantly differential metabolites between the two monoculture groups. (**E**) Hierarchical clustering heatmap of significantly differential metabolites between CWP under physical contact-free co-culture at a 1:5 ratio and CWP under monoculture. (**F**) Hierarchical clustering heatmap of significantly differential metabolites between CWP under physical contact-free co-culture at a 1:10 ratio and CWP under monoculture.

PCA revealed tight clustering within each CWP metabolome group and clear separation between the groups, indicating high intra-group reproducibility and significant inter-group metabolic differences (Figure S3). Under monoculture conditions, to support biomass accumulation, CWP significantly enriched metabolic pathways such as ABC transporters, protein digestion and absorption, and biosynthesis of amino acids (Figure 4A). In the low-inoculation-concentration monoculture of CWP, metabolites including asparagine, L-methionine, and palmitic acid were significantly enriched (Figure 4D), indicating substantial consumption of amino acids and fatty acids during biomass accumulation. These findings were consistent with the transcriptomic analysis (Figure 3A), collectively suggesting that CWP prioritizes amino acid metabolism during the early growth phase, followed by fatty acid synthesis and metabolism (Figure 3B,C). In contrast to monoculture, CWP under physical contact-free co-culture at a 1:5 ratio showed markedly active nucleotide metabolism and purine metabolism (Figure 4B). Moreover, the co-culture broth contained L-methionine, isovaleric acid, and other amino acids and fatty acids (Figure 4E), which may provide nutrients for the rapid growth of CWP. At the other inoculation ratio, CWP showed markedly active lysine biosynthesis and degradation metabolism (Figure 4C). Additionally, the co-culture broth contained palmitic acid and other fatty acids, along with TCA cycle intermediates such as succinate and cis-aconitate (Figure 4D). These results indicated that the co-culture broth was rich in various nutrients, which CWP may utilize to achieve biomass accumulation.

#### 3.4.2. Metabolomics Analysis of Y61

**Figure 5:**
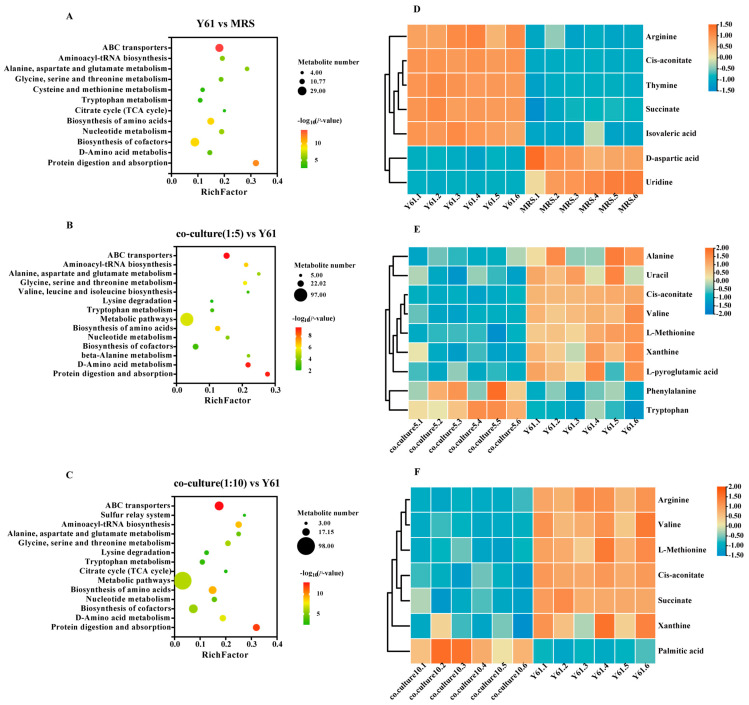
Metabolomics analysis results of Y61. (**A**) Bubble chart of significantly enriched pathways identified by KEGG analysis of differential metabolites between Y61 culture supernatant in MRS broth and MRS broth alone. (**B**) Bubble chart of significantly enriched pathways identified by KEGG analysis of differential metabolites between Y61 under physical contact-free co-culture at a 1:5 ratio and Y61 under monoculture. (**C**) Bubble chart of significantly enriched pathways identified by KEGG analysis of differential metabolites between Y61 under physical contact-free co-culture at a 1:10 ratio and Y61 under monoculture. (**D**) Hierarchical clustering heatmap of significantly differential metabolites between Y61 culture supernatant in MRS broth and MRS broth alone. (**E**) Hierarchical clustering heatmap of significantly differential metabolites between Y61 under physical contact-free co-culture at a 1:5 ratio and Y61 under monoculture. (**F**) Hierarchical clustering heatmap of significantly differential metabolites between Y61 under physical contact-free co-culture at a 1:10 ratio and Y61 under monoculture.

PCA results showed that samples within the same CWP metabolome group clustered tightly, while distinct separation was observed between the compared groups. This indicates high intra-group reproducibility and significant inter-group differences (Figure S4). As shown in Figure 5A, during growth in MRS broth, Y61 exhibited significant enrichment of pathways involved in ABC transporters, protein digestion and absorption, and amino acid biosynthesis. Furthermore, amino acids, fatty acids, and TCA cycle intermediates were significantly enriched in the culture broth (Figure 5D). Collectively, these findings demonstrated that Y61 could not only sustain normal growth and metabolism in MRS broth but also secrete various metabolites into the surrounding environment. In contrast to monoculture, Y61 under physical contact-free co-culture at both ratios exhibited persistently high activities of ABC transporters, protein digestion and absorption, and amino acid biosynthesis pathways (Figure 5B,C). This was accompanied by continuous secretion of metabolites such as arginine, succinate, and isovaleric acid into the extracellular space (Figure 5E,F). Notably, these nutrients did not accumulate in the co-culture medium. These results indicated that under co-culture conditions, Y61 exhibited active metabolism and continuously secreted nutrients, including amino acids and isovaleric acid, into the extracellular environment. These metabolites diffused upward through the 0.4 μm porous membrane separating the two chambers and may be directly utilized by CWP in the upper chamber, thereby supporting its metabolism and enabling rapid biomass accumulation.

### 3.5. Validation of Arginine and Isovaleric Acid

**Figure 6:**
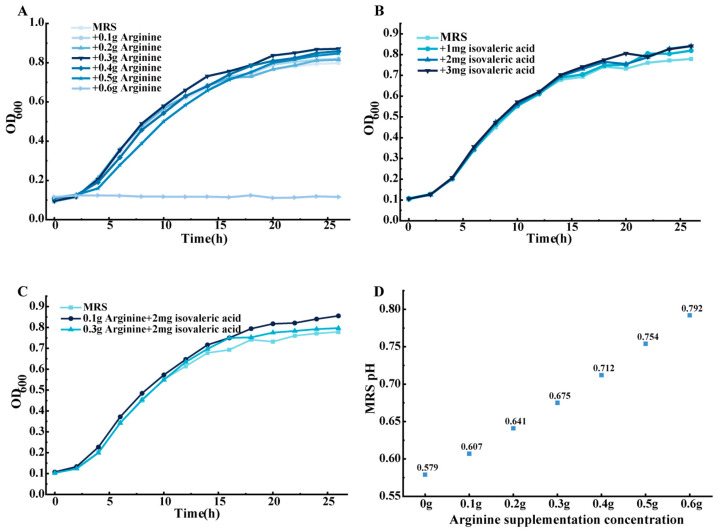
Growth curves of CWP under exogenous supplementation of arginine and/or isovaleric acid. (**A**) CWP growth with varying concentrations of arginine alone. (**B**) CWP growth with varying concentrations of isovaleric acid alone. (**C**) CWP growth with combined supplementation of arginine and isovaleric acid at selected doses. (**D**) Effect of arginine supplementation concentration on the pH of MRS broth.

Based on the transcriptomic and metabolomic results described above, Y61 promoted CWP growth by secreting the key metabolites arginine and isovaleric acid. To verify whether these two metabolites indeed exerted this effect, we exogenously supplemented arginine and isovaleric acid into MRS broth, cultured CWP in this modified medium, and examined whether its growth was altered. Exogenous arginine promoted CWP biomass accumulation at supplementation levels below 0.4 g, with optimal promotion at 0.3 g. The promoting effect weakened when arginine exceeded 0.4 g, and CWP failed to grow at 0.6 g (Figure 6A). The underlying reason was that each 0.1 g of arginine supplementation raised the pH of MRS broth by 0.3–0.4, and the excessive pH inhibited CWP growth (Figure 6D). Exogenous isovaleric acid at all three tested concentrations promoted CWP biomass accumulation, with the 2 mg and 3 mg supplementation groups showing similar CWP biomass levels after 26 h of cultivation (Figure 6B). The optimal doses from individual supplementation were selected for combined addition. The combination of 0.1 g arginine and 2 mg isovaleric acid showed the strongest promotion of CWP growth. Additionally, combining the optimal individual arginine dose (0.3 g) with 2 mg isovaleric acid also enhanced CWP growth (Figure 6C).

## 4. Discussion

A close relationship exists between the compositional differences in bacterial communities and the flavor quality as well as physicochemical properties of paocai [32]. Our previous study revealed that Y61 supplementation significantly increased the abundance of *Weissella* at the early stage of paocai fermentation. Building on this finding, the present study further investigated the underlying mechanism of Y61-promoted *Weissella* growth at the culture medium level. In this study, we first cultured *Weissella* CWP with CFS of Y61, which preliminarily confirmed the existence of a promoting relationship between these two strains. To further elucidate the mechanism underlying this promoting effect, a transwell chamber was used to physically separate Y61 and CWP during co-culture. Subsequently, transcriptomic and untargeted metabolomic analyses were performed to investigate the material basis of cross-feeding. After identifying key metabolites, their functions were validated through exogenous supplementation experiments.

In this study, the OD_600_ value was used as an indicator of microbial biomass accumulation to assess the growth rate of CWP. The results showed that the OD_600_ value of CWP cultured in Y61 CFS was higher than that in MRS broth (Figure 2A), indicating that Y61 cell-free supernatant promoted CWP growth more effectively. Furthermore, the pH value of the Y61 CFS culture was slightly lower than that of MRS broth (Figure 2B). This indicated that Y61 secreted metabolites into the extracellular environment during its growth, which in turn promoted the growth of CWP. Metabolic exchange among microbial populations facilitated interspecies interactions. When the metabolites or extracellular compounds produced by one species are utilized as metabolic substrates by another species, this phenomenon is referred to as cross-feeding or metabolic cooperation [33]. To elucidate the feeding-promoting effect of Y61 on CWP, a physical contact-free co-culture system was employed. A transwell insert equipped with a 0.4 μm pore membrane was used to physically separate Y61 and CWP while permitting the free exchange of metabolites between the upper and lower chambers. Paocai fermentation primarily relies on the action of lactic acid bacteria and yeasts [34]. Therefore, to mimic the relative abundance of Y61 and CWP during the early phase of paocai fermentation, two initial inoculation ratios, namely Y61:CWP = 1:5 and Y61:CWP = 1:10, were established. Figure 2C,D show the changes in OD_600_ values of the two strains in the upper and lower chambers after 48 h of cultivation. A limitation of the transwell system is evaporation in the upper chamber during prolonged culture (>36 h), which caused artifactual OD_600_ readings at 48 h. Therefore, this 48 h time point was excluded from the final analysis. The results demonstrated that when CWP was inoculated in the upper chamber and Y61 in the lower chamber, CWP exhibited a more pronounced increase in OD_600_ value and greater biomass accumulation (Table 1 and Table S2). Microaerobic conditions are more favorable for maintaining the original microbial community of paocai, whereas under anaerobic conditions, lactic acid bacteria still dominate [35]. This finding suggested that metabolites secreted by Y61 in the lower chamber diffuse through the membrane into the upper chamber, where they might be directly utilized by CWP, thereby accelerating its growth.

Transcriptomics enables the systematic analysis of the entire collection of transcripts (mRNA) expressed by an organism under specific physiological or environmental conditions [36]. Transcriptomic analysis reveals the differentially expressed genes and associated metabolic pathways involved under different inoculation concentrations and culture conditions. Under monoculture conditions, compared with the higher inoculation concentration (CWP10), the lower inoculation concentration (CWP5) significantly upregulated genes involved in amino acid metabolism, including PLP-dependent transferase and homoserine O-succinyltransferase (Figure 3A). These differentially expressed genes were significantly enriched in the ABC transporters and cysteine and methionine metabolism pathways (Figure 3D); the genome of *Weissella* harbors a large number of genes involved in carbohydrate metabolism, environmental stress adaptation, and membrane transport [37], which conferred upon this bacterium the ability to efficiently uptake and utilize extracellular nutrients. Compared with CWP under monoculture at the same inoculation concentration, CWP under physical contact-free co-culture exhibited significantly enhanced pathways related to fatty acid synthesis and metabolism (Figure 3E,F); genes involved in fatty acid synthesis and metabolism, such as *fabG*, were significantly upregulated (Figure 3B,C). *Weissella* exhibits selective enrichment in environments containing short-chain fatty acids such as acetic acid [38], indicating its metabolic preference for short-chain fatty acids and its ability to utilize them extensively. These results indicated that under monoculture conditions, CWP primarily engaged in amino acid synthesis and metabolism to build macromolecular backbones, thereby achieving biomass accumulation. In contrast, during co-culture with Y61, the fatty acid synthesis and metabolism pathways in CWP were significantly activated. This observation suggested that Y61 may secrete fatty acids into the co-culture system, which are then directly metabolized and utilized by CWP for its own fatty acid synthesis.

Metabolomics enables comprehensive analysis of small-molecule metabolites in a biological culture system, thereby directly revealing microbial metabolic activities, physiological states, and metabolic interactions among different species within the culture system [39]. Metabolomics analysis was performed on the monoculture and co-culture fermentation supernatants of Y61 and CWP. These results showed that under monoculture conditions, as CWP biomass accumulated, asparagine, L-methionine, and palmitic acid were significantly consumed in the culture system (Figure 4D). Meanwhile, metabolic pathways including ABC transporters, biosynthesis of amino acids, and protein digestion and absorption were significantly active in CWP (Figure 4A). Under co-culture conditions, compared with monoculture, the co-culture fermentation supernatant was significantly enriched with metabolites including isovaleric acid, L-methionine, and cis-aconitate (Figure 4E,F). These differential metabolites were mainly enriched in metabolic pathways such as protein digestion and absorption, D-amino acid metabolism, nucleotide metabolism, and biosynthesis of amino acids (Figure 4B,C). These metabolomic results were highly consistent with the transcriptomic analysis (Figure 3A–C). The significant enrichment of metabolites such as isovaleric acid, L-methionine, and cis-aconitate in the co-culture fermentation supernatant provided abundant metabolic substrates for CWP growth. The metabolomic results from Y61 cultured in MRS broth also provided strong support for this conclusion. In contrast to un-inoculated MRS broth, the MRS broth conditioned by Y61 growth exhibited significant enrichment of arginine, isovaleric acid, and succinate (Figure 5D). This finding demonstrated that the prominent differential metabolites observed in the co-culture supernatant were derived from Y61 secretion. Compared with the Y61 monoculture supernatant, metabolites including arginine, isovaleric acid, succinate, and cis-aconitate were significantly depleted in the co-culture fermentation supernatant (Figure 5E,F). This indicated that under co-culture conditions, these metabolites secreted by Y61 might be taken up and metabolically consumed by CWP as substrates.

Previous studies have shown that free amino acids in fruit and vegetable puree can be utilized by lactic acid bacteria for fermentation, leading to the production of flavor compounds. Furthermore, exogenous supplementation of amino acids can enrich the diversity of flavor compounds produced during lactic acid bacteria fermentation [40]. Additional studies have shown that increased fatty acid content significantly promotes the accumulation of *Weissella*. The genus *Weissella* was significantly enriched in the gut of vegetarians, which was characterized by a higher abundance of short-chain fatty acids and amino acids [41]. Grass carp fed an artificial diet exhibit enhanced lipid metabolism and increased biosynthesis of fatty acids, glycerol, and glycerophospholipids, and their gut microbiota is dominated by *Weissella* and *Cetobacterium somerae* [42]. Under conditions without pH adjustment, exogenous arginine and isovaleric acid were found to promote CWP growth. This finding agreed well with earlier studies. OD_600_ measurements over a 26 h cultivation period revealed that the most significant growth promotion was achieved with 0.3 g arginine or 2 mg isovaleric acid when added individually (Figure 6A,B). When the two compounds were added in combination, the greatest synergistic effect was observed with 0.1 g arginine and 2 mg isovaleric acid (Figure 6C). This observation can be explained by two considerations. On one hand, 0.1 g arginine exerted a minimal effect on the pH of MRS broth, preventing the suppression of CWP growth due to excessively high pH (Figure 6D). On the other hand, arginine and isovaleric acid likely underwent an acid-base neutralization reaction, leading to their predominant existence as ion pairs, a form that was more accessible for CWP uptake and metabolism. But this proposed mechanism remains speculative and requires further validation through targeted experiments, such as ion pair detection or cellular uptake assays, in future studies. It should be noted that the concentrations of arginine and isovaleric acid employed in this study were based on literature data rather than precise pre-experimental quantification. Nevertheless, future investigations should include targeted quantitative analyses to obtain more accurate concentration determinations of these two compounds.

In summary, using a transwell co-culture system combined with transcriptomic and metabolomic analyses, this study elucidated the mechanism by which Y61 promotes CWP growth. Y61 could secrete the key metabolites arginine and isovaleric acid into the extracellular environment during its metabolism, and CWP may directly take up and utilize these metabolites to achieve rapid growth. It should be noted that the conclusions of this study were drawn from a simplified model using MRS broth and were not validated in the complex native paocai system. Therefore, the relevance of this mechanism in the actual paocai ecosystem remains to be further investigated.

## 5. Conclusions

In this study, we systematically elucidated the interaction mechanism between *Bacillus subtilis* Y61 and *Weissella paramesenteroides* CWP using CFS assays, transwell co-culture experiments, and integrated transcriptomic and untargeted metabolomic analyses. The findings demonstrated that Y61 could promote the growth of CWP in a contact-independent manner, suggesting that diffusible metabolites played a central role in mediating interspecies interactions. Multi-omics analyses further revealed that co-culture with Y61 induced substantial metabolic reprogramming in CWP, particularly in pathways associated with amino acid utilization and fatty acid biosynthesis, indicating enhanced metabolic activity and environmental adaptation during co-culture. In addition, metabolite profiling combined with supplementation experiments identified arginine and isovaleric acid as key metabolites involved in this cooperative interaction. These results suggested that metabolic cross-feeding constituted an important ecological strategy underlying the coexistence and succession of microorganisms in the paocai fermentation ecosystem. Overall, this study provided new insights into the molecular basis of microbial cooperation in fermented vegetables and offered a theoretical foundation for the rational design of synthetic microbial consortia to achieve controlled paocai fermentation with improved quality and safety.

## Figures and Tables

**Table 1:** Colony counts of CWP in mono-culture and co-culture at 36 h.

Ratio	Group	CFU Counts
Y61:CWP = 1:5	CWP-up	5.6 × 10^9^ CFU/mL
Y61:CWP = 1:5	CWP-down	1.1 × 10^9^ CFU/mL
Y61:CWP = 1:5	co-culture	5.0 × 10^8^ CFU/mL
Y61:CWP = 1:10	CWP-up	2.4 × 10^9^ CFU/mL
Y61:CWP = 1:10	CWP-down	8.0 × 10^8^ CFU/mL
Y61:CWP = 1:10	co-culture	6.0 × 10^8^ CFU/mL

## Data Availability

The original contributions presented in this study are included in the article and Supplementary Materials. Further inquiries can be directed to the corresponding author.

## Supplementary Materials

The following supporting information can be downloaded at https://www.mdpi.com/article/10.3390/foods15132266/s1.
