# Supplementary material for: Mechanism of Bacillus subtilis Y61 Promoting the Growth of Weissella: Metabolic Interaction Based on Secretion of Arginine and Isovaleric Acid
Source: Foods. 2026 Jun 24;15(13):2266. doi: 10.3390/foods15132266 (PMC13360752; doi:10.3390/foods15132266)
Supplement: Supplementary file 1 [file foods-15-02266-s001.zip › foods-4364327-supplementary.pdf]

Table S1. RIN of CWP transcriptome.

Sample name	RIN	Assay Conclusion
CWP-up(1:5)-1	9.9	A
CWP-up(1:5)-2	9.9	A
CWP-up(1:5)-3	9.9	A
CWP-up(1:10)-1	9	A
CWP-up(1:10)-2	9.4	A
CWP-up(1:10)-3	9.6	A
CWP (5)-1	9.8	A
CWP (5)-2	9.5	A
CWP (5)-3	9.8	A
CWP (10)-1	8.7	A
CWP (10)-2	9.3	A
CWP (10)-3	8.9	A

Table S2. Colony counts of CWP in mono-culture and co-culture at 12h, 24h.

Time	Ratio	Group	CFU counts
12h	Y61: CWP=1:5	CWP-up	9.8×10^{8}CFU/mL
	Y61: CWP=1:5	CWP-down	9×10^{8}CFU/mL
	Y61: CWP=1:5	co-culture	6.44×10^{8}CFU/mL
	Y61: CWP=1:10	CWP-up	9×10^{8}CFU/mL
	Y61: CWP=1:10	CWP-down	1.44×10^{8}CFU/mL
	Y61: CWP=1:10	co-culture	3.88×10^{8}CFU/mL
24h	Y61: CWP=1:5	CWP-up	1.44×10^{9}CFU/mL
	Y61: CWP=1:5	CWP-down	1.03×10^{9}CFU/mL
	Y61: CWP=1:5	co-culture	6.96×10^{8}CFU/mL
	Y61: CWP=1:10	CWP-up	1.54×10^{9}CFU/mL
	Y61: CWP=1:10	CWP-down	5.01×10^{8}CFU/mL
	Y61: CWP=1:10	co-culture	4.86×10^{8}CFU/mL

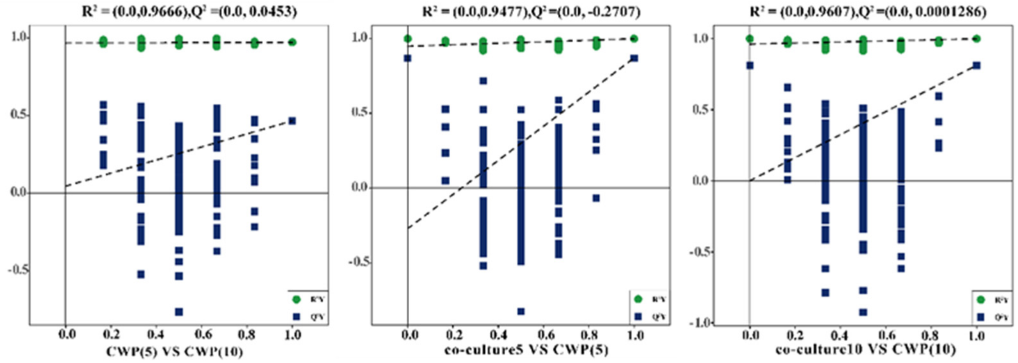

Figure S1. Permutation test of the OPLS-DA model for the CWP untargeted metabolome.

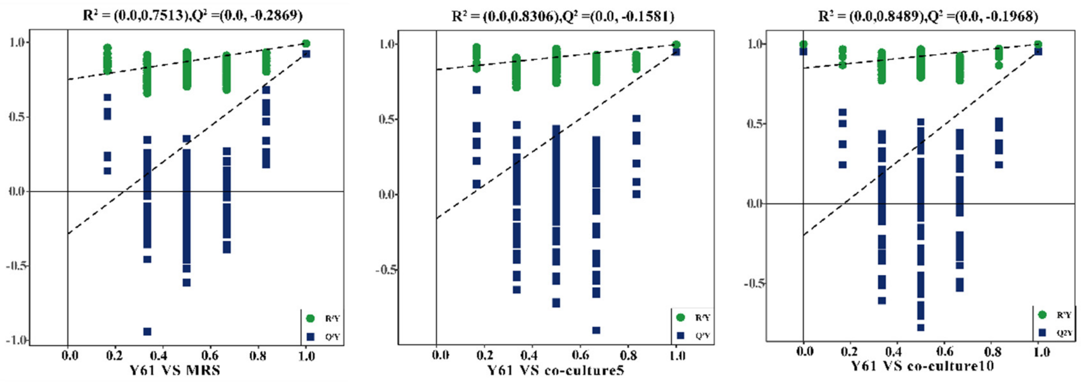

Figure S2. Permutation test of the OPLS-DA model for the Y61 untargeted metabolome.

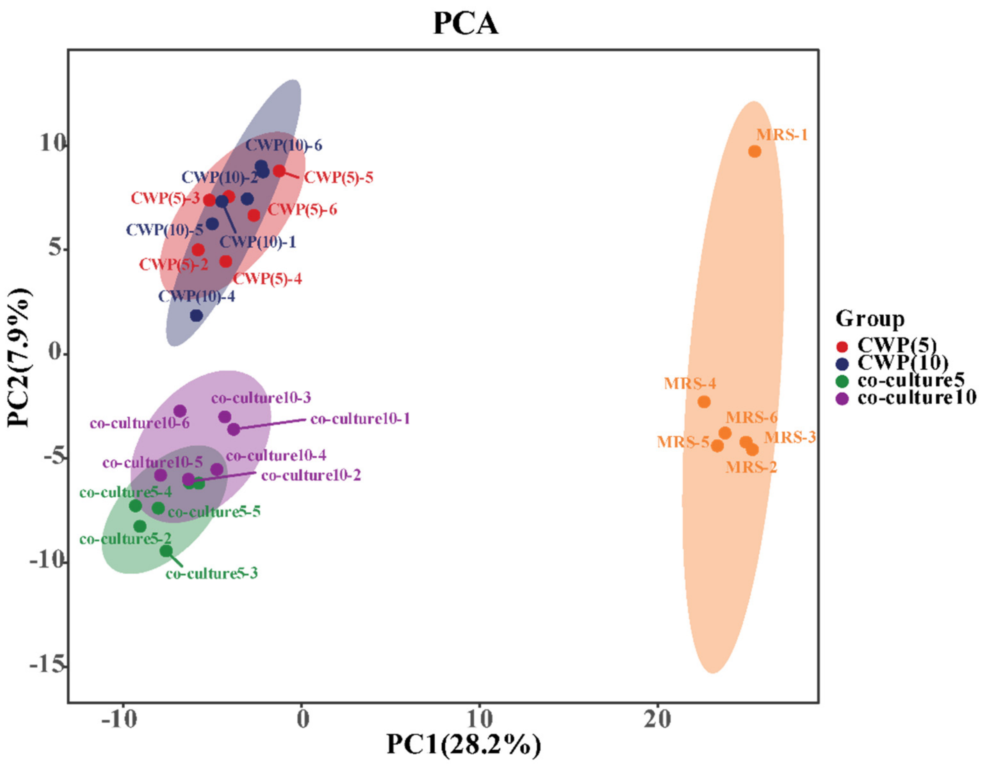

Figure S3. PCA clustering based on CWP untargeted metabolomics.

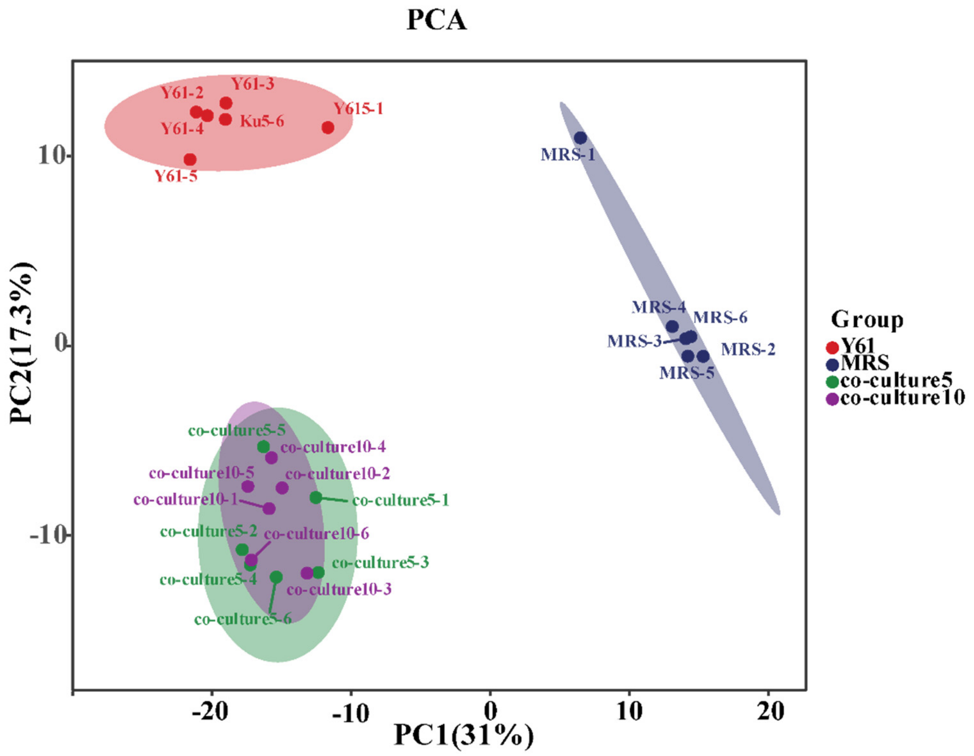

Figure S4. PCA clustering based on Y61 untargeted metabolomics.
